# 

**Published:** 1974-04

**Authors:** 


					IS
MEDICO
CHIRURGICAL
JOURNAL
U
? L s&S&JSSfia
, j i - I - wn
1? TMjz I '
?7sen
Arji
I
^ PT~ c. ,pp,
^OKDEXMEfilCUj
APRIL 1974
VOL. 89 (ii) No. 330

				

